# Comparison of Early Efficacy of the Percutaneous Presuture Technique with the Femoral Artery Incision Technique in Endovascular Aortic Repair under Local Anesthesia for Uncomplicated Type B Aortic Dissection

**DOI:** 10.1155/2022/6550759

**Published:** 2022-08-22

**Authors:** Qingsong Wu, Debin Jiang, Xiaochai Lv, Jiaxin Zhang, Rongda Huang, Zhihuang Qiu, Liangwan Chen

**Affiliations:** ^1^Department of Cardiovascular Surgery, Union Hospital, Fujian Medical University, Fuzhou, Fujian 350001, China; ^2^Fujian Key Laboratory of Cardio-Thoracic Surgery, Fujian Medical University, Fuzhou, Fujian 350001, China; ^3^Fujian Provincial Special Reserve Talents Laboratory, Fuzhou, Fujian 350001, China; ^4^Engineering Research Center of Tissue and Organ Regeneration, Fujian Province University, Fuzhou, Fujian 350001, China; ^5^Fujian Key Laboratory of Vascular Aging, Fujian Medical University, Fuzhou, Fujian 350001, China

## Abstract

**Objective:**

To compare the efficacy of the percutaneous presuture technique (PPST) and the femoral artery incision technique (FAIT) under local anesthesia in the treatment of endovascular aortic repair (EVAR) for patients with uncomplicated type B aortic dissection (uTBAD).

**Method:**

Two hundred and ninety-five patients diagnosed with uTBAD who underwent EVAR under local anesthesia from June 2017 to December 2021 were consecutively and randomly selected for retrospective analysis. The PPST was performed in 178 cases and the FAIT was performed in 117 cases. The clinical characteristics and surgical and postoperative data from the two groups were analyzed.

**Results:**

There were no significant differences in clinical characteristics between the two groups (*p* > 0.05). The operative time of the PPST group was significantly shorter than that of the FAIT group (46 (33, 58) versus 72 (67.5, 78.0) minutes, *p* < 0.001), as was the operative approach procedure time (6 (4.5, 9.0) versus 38 (36.5, 43.5) minutes, *p* < 0.001), and length of postoperative hospital stay (5.19 ± 2.26 versus 8.33 ± 3.76 days, *p* < 0.001). There were fewer postoperative approach-related procedural complications in the PPST group than in the FAIT group (2 versus 12, *p* < 0.001); similarly, the average frequency of postoperative wound disinfection was significantly lower in the PPST group (1.08 ± 0.39 versus 3.31 ± 0.91 times, *p* < 0.05). Obesity was identified as an independent risk factor for postoperative approach-related procedural complications (OR, 22.26; 95% CI, 4.74–104.49; *p* < 0.001).

**Conclusions:**

The PPST has comparable safety and efficacy to the FAIT in EVAR under local anesthesia. It can shorten the length of hospital stay, reduce operation time, lower the risk of wound-related complications, reduce the frequency of postoperative wound disinfection, and hasten postoperative recovery. It can therefore be used as a first-line surgical technique in EVAR of uTBAD under local anesthesia, especially in obese patients.

## 1. Introduction

Since the development of minimally invasive interventional technology over the last 20 years, endovascular aortic repair (EVAR) has become the preferred approach in the surgical management of Stanford type B aortic dissection (TBAD). It has many advantages over the traditional operative approach, as it induces less trauma and offers a better safety profile and a faster postoperative recovery time [1–3]. EVAR for uncomplicated TBAD (uTBAD) can usually be performed under local anesthesia [4]. The traditional operative approach requires incision, exposure, and suturing of the femoral artery puncture site, which is time-consuming and causes patient discomfort during incision of the femoral artery. It can also damage the surrounding tissue, blood vessels, lymphatic system, and nerves. Postoperative complications such as hematoma, infection of the incisional site, lymphocele of the surgical incision, vascular injury, arteriovenous fistula, paresthesia of the skin around the incision (femoral neuropathy), pseudoaneurysm, poor incisional healing, and femoral stenosis, thrombosis, or occlusion commonly occur. This not only prolongs the length of hospital stay but also impacts the postoperative quality of life of patients. The percutaneous presuture technique (PPST) allows EVAR to be even less invasive. The PPST creates embedded sutures and avoids femoral artery incisions for most EVAR patients. It inflicts minimal damage to the tissue surrounding the femoral artery, which reduces the length of surgery and the incidence of local incisional complications [5, 6].

This study aimed to retrospectively investigate the differences in operation time, length of hospital stay, postoperative wound-related complications, and other early postoperative complications between the PPST and the FAIT in EVAR under local anesthesia for patients with uTBAD.

## 2. Patients and Methods

### 2.1. Study Population

**Figure 1:**
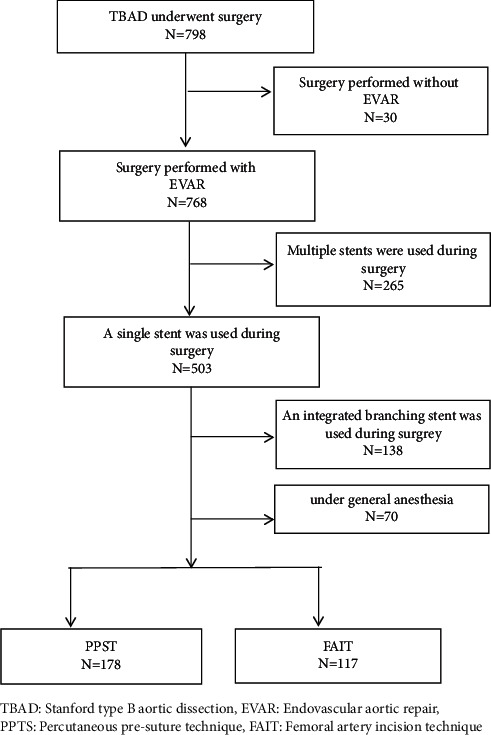
A consort type diagram of whole patients with TBAD who underwent surgery.

This retrospective study included patients who presented to our hospital between June 2017 and October 2021. We included patients who (1) had a diagnosis of uTBAD as confirmed by magnetic resonance angiography or computed tomography angiography, (2) had undergone EVAR under local anesthesia, and (3) had only 1 stent placed intraoperatively, with no branching stents. We excluded patients if they had (1) previous bilateral femoral artery or vein surgery, (2) diagnosis of complicated TBAD and Stanford type A aortic dissection, or (3) cachexia associated with malignancy (Figure 1).

This study conformed to the Declaration of Helsinki and was approved by the ethics committee of Union Hospital, Fujian Medical University. In accordance with national guidelines, this retrospective review of patient data did not require written informed consent from participants.

### 2.2. Clinical Data

The data collected included patient baseline characteristics, operative details, and early postoperative outcomes. Baseline characteristics included information on sex, age, obesity, hypertension, diabetes mellitus, coronary heart disease, hypercholesterolemia, hypoalbuminemia, moderate anemia, platelet count, white blood cell count, and the anatomical features of the femoral arteries. Operative details included operative approach, procedure time, total operative time, intraoperative blood loss, number of Perclose ProGlide suture-mediated systems® (Abbott Vascular Corp, CA, USA) used, stent information, and sheath diameter. Early postoperative outcomes included length of hospital stay and procedure-related complications associated with the operative approach, including percutaneous puncture failure, arteriovenous fistula, femoral neuropathy, hematoma, hemorrhage, infection, poor wound healing, lymphocele, thrombosis/occlusion, vascular injury, pseudoaneurysm, lower limb ischemia, and femoral artery dissection. The uTBAD was defined as type B aortic dissection in which patients were not associated with malperfusion (which could result in end-organ ischemia and severely affect an organ or limb perfusion), periaortic hematoma with blood collection, persistent hypertension despite full medical therapy, hemorrhagic pleural effusion, or aortic rupture. Percutaneous puncture failure was defined as transfer or surgical intervention due to complications at the puncture site. The use of more than four devices to complete suturing was also considered a failure. Successful closure was defined as the successful completion of the femoral artery puncture site closure without additional surgical or other interventions. Hypoproteinemia was defined as serum albumin levels < 30 g/L. Moderate anemia was defined as hemoglobin levels < 90 × 10^9^ g/L. Hypercholesterolemia referred to an increase in total cholesterol and/or low-density lipoprotein cholesterol or non-high-density lipoprotein cholesterol in the blood. Obesity was defined as body mass index (BMI) > 30 kg/m^2^.

### 2.3. PPST

All instances of the PPST were performed by our surgical team with the use of the Perclose ProGlide suture-mediated system. All procedures were performed under local anesthesia using lidocaine. The Seldinger technique was used to puncture the femoral artery, and heparin (1 mg/kg) was used for anticoagulation. The Perclose ProGlide suture-mediated system was used for preimplantation. After standard EVAR, the preimplantation Perclose ProGlide suture-mediated system was tied to control bleeding, the tissue around the incision was massaged, and pressure was applied manually for 5 minutes. If any bleeding was noted, further compression was applied until there was no bleeding at the puncture point, and appropriate compression dressings were then applied. The dorsalis pedis artery pulse in the right lower limb was palpated at the conclusion of surgery.

### 2.4. FAIT

A femoral artery incision was performed under local anesthesia, and the strongest pulse point of the femoral artery approximately 1 cm above the inguinal ligament was selected as the area of the incision. A 4 cm oblique incision was made, the subcutaneous tissue, fat, and fascia were separated, the vascular sheath was opened, and the common femoral artery was isolated. A 5–0 prolene suture was used to create two pockets in the common femoral artery. The femoral artery was punctured directly in the middle of the purse created using the Seldinger technique. Heparin (1 mg/kg) was administered for anticoagulation. After EVAR was successfully performed, the intra-arterial sheath tube was removed and the distal and proximal blood flow at the puncture site was temporarily paused. Continuous horizontal mattress valgus sutures were completed with 5–0 prolene. The distal and proximal blocking forceps were then successively loosened, gas in the vessels of the sutured segment was drained, unobstructed blood flow was ensured, and the incision was knotted and closed layer by layer.

### 2.5. Statistical Analysis

All statistical analyses were performed using SPSS version 19.0 (SPSS Inc, Chicago, IL, USA). Categorical variables were presented as numbers and percentages (*n*, %), continuous variables were presented as means ± standard deviations, and variables without normal distributions were expressed as medians with interquartile ranges. The *t*-test and *χ*^2^ test were used for categorical variables, and the Wilcoxon rank‐sum test was used for continuous variables. Univariable logistic regression analysis was used to identify potential risk factors for poor postoperative outcomes. Variables with *p* < 0.200 in the univariable model were entered into the multivariable model. The threshold for statistical significance was set at *p* < 0.05.

## 3. Results

There were no significant differences in age, sex, obesity, hypertension, diabetes, coronary heart disease, hyperlipidemia, hypoproteinemia, moderate anemia, platelet count, white blood cell count, anatomical features of the femoral arteries, and other risk factors between the two groups (*p* > 0.05) (Table 1).

Patients in both groups were successfully operated on under local anesthesia. In the PPST group, the success rate of percutaneous puncture was 100%, and the average number of Perclose ProGlide suture-mediated systems used was 2.12 ± 0.39. The femoral artery was successfully closed postoperatively. In the FAIT group, the femoral artery was sutured successfully without tears or dissection. The operative time of the PPST group was significantly shorter than that of the FAIT group (46 (33, 58) versus 72 (67, 81) minutes, *p* < 0.001). The operative time of the PPST procedural approach was shorter than that of the FAIT (6 (4.5, 9.0) versus 38 (36, 45) minutes, *p* < 0.001). There was no statistical difference in the amount of intraoperative blood loss between the two groups (Table 2).

The length of postoperative hospital stay in the PPST group was significantly shorter than that of the FAIT group (5.19 ± 2.26 versus 8.33 ± 3.76 days, *p* < 0.001), and postoperative approach-related procedural complications in the PPST group were less than that in the FAIT group (2 versus 12, *p* < 0.001). In the PPST group, there was 1 case of hematoma and 1 case of arteriovenous fistula, while in the FAIT group, there were 3 cases of lymphocele, 2 cases of poor wound healing, 1 case of infection, 2 cases of vascular injury, 2 cases of femoral neuropathy, 1 case of hemorrhage, and 1 case of hematoma. The average frequency of postoperative incisional disinfection in the PPST group was significantly lower than that in the FAIT group (1.08 ± 0.39 versus 3.31 ± 0.91, *p* < 0.001) (Table 3).

Univariable analysis suggested that age, obesity, hypertension, coronary heart disease, hypercholesterolemia, hypoproteinemia, operative time, operative approach procedure time, a sheath diameter of 16 Fr, and group (PPST or FAIT) were potential risk factors for postoperative approach-related procedural complications. After adjusting for these factors, obesity was identified as the independent risk factor for postoperative approach-related procedural complications (OR, 22.26; 95% CI, 4.74–104.49; *p* < 0.001) (Table 4).

## 4. Discussion

EVAR has supplanted open aortic replacement as the surgical treatment of choice for most TBAD patients [7–13]. It has obvious advantages. Most simple endovascular stent implantations at our center can be performed under local anesthesia, which greatly reduces the use of ventilators, ventilator-related complications, and the length of hospital stay. Traditional EVAR requires a femoral artery incision, which not only creates an incisional wound but also prolongs the operation time. Moreover, the incidence of postoperative complications is high and there is a relatively long recovery time. The consequent impact on the average duration of hospital stay has become an issue in China. This is not in line with China's current policy of reducing the average hospitalization duration for patients. Reducing operation times and postoperative hospitalization duration can improve the utilization rate of operating theaters, increase hospital bed turnover, relieve pressure on the healthcare system, and consequently confer economic benefits. EVAR combined with the PPST under local anesthesia not only simplifies the operative approach but also greatly shortens the operation time and improves operative efficiency. Minimally invasive wound management also improves the quality of life of patients [14–17].

The success rate of the PPST was 100%, suggesting that this is a simple and safe procedure that does not introduce additional surgery-related risks. We, therefore, propose that it may be widely adopted in EVAR. Studies have shown that this technique is relatively simple to learn [18], and has been mastered in a short amount of time by surgeons at our center. The PPST can therefore be used as a safe and effective alternative to the FAIT under local anesthesia. Although the PPST has a high success rate, there is still the possibility of puncture failure or even the need for transfer surgery. As such, the PPST should be performed by experienced vascular surgeons.

In this study, there was only 1 case of arteriovenous fistula and 1 case of hematoma in the PPST group, which were resolved for both patients. Both patients were obese with a BMI of 31.4 and 33.8, respectively. Due to the thick subcutaneous fat layer, the pulse of the common femoral artery was difficult to isolate. The puncture path was also longer than average, necessitating the need for an ultrasound-guided puncture. In these patients, the Perclose ProGlide suture-mediated system was tied and the puncture site was kneaded before a sterile gauze and compression dressing were applied. We conclude that ultrasound-guided puncture is recommended for obese patients, and local tissue kneading and compression dressing can help to support the puncture wound.

The diameter of the delivery tube sheath, calcification of the femoral artery, and anatomical morphology of the femoral artery are also important factors affecting the success of surgery [19–21]. In this study, no patients with femoral artery puncture needed to be transferred for incision. Georgiadis et al. published a meta-analysis demonstrating that the outer diameter of the conveyor sheath was significantly associated with the need for surgical transfer [OR, 1.78; 95% CI, 1.24–2.54] and that the risk of suture failure requiring surgical management was significantly higher if the outer conveyor sheath was ≥ 20 Fr in diameter compared with a diameter ≤18 Fr [19]. Jaffan et al. concluded that the technical success rate of the ≥ 20 Fr diameter group was lower than that of the ≤18 Fr diameter group (88.7% versus 94.2%, *p*=0.0001) [22]. However, there are reports that there is no correlation between the outer diameter of the conveyor sheath tube and failure of surgery [23]. Since all patients in our study were implanted with a single stent, and the size of the vascular sheath was between 16 and 20 Fr, there were no instances of intraoperative implantation of two different stents in the vascular sheath. Therefore, further research is needed on the correlations between the size of the vascular sheath used in the PPST and the implantation of multiple stents.

In previous studies, the incidence of postoperative short-term complications of percutaneous EVAR was lower than that of traditional femoral artery incision [24, 25], which is similar to the results of this study. We did not observe any medium- or long-term complications of the PPST, but relevant studies from other countries suggest that these rates after percutaneous EVAR are relatively low [26, 27].

The PPST has the advantage of embedded sutures, which enables most EVAR patients to avoid femoral artery incision and means that it can be performed under local anesthesia, thus reducing the incidence of local incisional complications [28, 29]. All included patients underwent surgery under local anesthesia, thus eliminating the need for endotracheal intubation and avoiding the risk of ventilator-associated pneumonia. This reduces the need for resuscitation and postoperative bed rest time and omits other postoperative protocols that would otherwise be required. A wound drainage device is not required, nor do patients need to fast before surgery. These factors significantly decrease patient discomfort and improve the overall treatment experience.

In conclusion, our study demonstrates that the PPST in EVAR under local anesthesia has good short-term safety and effectiveness. It is an alternative to the traditional FAIT and reduces operation time, length of hospital stay, and operative approach-related procedural complications, and alleviates the burden on the healthcare system.

### 4.1. Limitations

Only patients with single stents were included in our study, and the stent and vascular sheath were not alternated during surgery. Prospective controlled studies with larger sample sizes are required to analyze the efficacy and safety of the PPST in the context of branching or multiple vascular stents.

## 5. Conclusions

In EVAR under local anesthesia, the PPST can reduce the postoperative duration of hospital stay, the operative approach procedure time, total operative time, operative approach-related procedural complications, frequency of postoperative wound disinfection, and postoperative recovery time compared with the FAIT. It can therefore be adopted as the preferred method for EVAR of uTBAD under local anesthesia, especially in obese patients.

## Figures and Tables

**Table 1:** Preoperative data on the two patient groups.

Valuables	PPST (*n* = 178)	FAIT (*n* = 117)	*p* value
Age (years)	59.0 (50.0, 68.0)	57.0 (53.5, 60.5)	0.064
Male gender (%)	141 (79.2)	97 (82.9)	0.433
Obesity (%)	10 (5.6)	8 (6.8)	0.669
Hypertension (%)	152 (85.4)	98 (83.8)	0.703
Diabetes mellitus (%)	5 (2.8)	5 (4.3)	0.521
Coronary heart disease (%)	6 (3.4)	7 (6.0)	0.286
Hypercholesterolemia (%)	18 (10.1)	17 (14.5)	0.252
Hypoproteinemia (%)	10 (5.6)	5 (4.3)	0.608
Moderate anemia (%)	7 (3.9)	3 (2.6)	0.694
Platelet count (^*∗*^10^9^/L)	187.5 (152.0, 228.0)	188.0 (158.5, 194.0)	0.313
White blood cell count (^*∗*^10^9^/L)	10.01 (8.35, 12.86)	8.70 (6.09, 11.02)	0.353
Anatomy features of the femoral arteries			
Calcification	4 (2.2)	7 (8.0)	0.183
Depth of femoral artery^*∗*^	41.0 (36.8, 52.1)	38.4 (32.6, 50.6)	0.085
Dissection involving	0 (0.0)	3 (2.6)	0.120
Diameter	8.3 (7.9, 8.8)	8.3 (7.9, 8.7)	0.267

Continuous variables were present as median (Q25, Q75). Wilcoxon rank test for continuous variables and *χ*^2^ test for categorical variables. ^*∗*^Axial computed tomography slice passing through the geometric center of the femoral heads, showing the plane of the ischial spine on both sides and the vertical distance from the central point of the common femoral artery to the skin surface.

**Table 2:** Surgical data on the two patient groups.

Valuables	PPST (*n* = 178)	FAIT (*n* = 117)	*p* value
Operative time (min)	46.0 (33.0, 58.0)	72.0 (67.5, 78.0)	<0.001
Operative approach procedure time (min)	6.0 (5.0, 9.0)	38.0 (36.5, 43.5)	<0.001
Intraoperative blood loss (mL)	25.0 (25.0, 35.0)	45.0 (45.0, 55.0)	0.176
Number of perclose ProGlide suture-mediated systems used	2.12 ± 0.39	N/A	N/A

Stent information			
Lifetech scientific (%)	143 (80.3)	96 (82.0)	0.713
Medtronic (%)	23 (12.9)	12 (10.3)	0.489
MicroPort (%)	12 (6.8)	9 (7.7)	0.756

Sheath diameter			
16Fr (%)	45 (25.3)	26 (22.2)	0.548
18Fr (%)	103 (57.9)	63 (53.9)	0.496
20Fr (%)	30 (16.8)	28 (23.9)	0.135

Continuous variables were present as median (Q25, Q75). *χ*^2^ test for categorical variables and Wilcoxon rank sum test for continuous variables.

**Table 3:** Postoperative data on the two patient groups.

Valuables	PPST (*n* = 178)	FAIT (*n* = 117)	*p* value
Postoperative approach procedure-related complications (%)	2 (1.1)	12 (10.3)	<0.001
Arteriovenous fistula (%)	1 (0.6)	0 (0.0)	N/A
Femoral neuropathy (%)	0 (0.0)	2 (1.7)	N/A
Hematoma (%)	1 (0.6)	1 (0.9)	0.764
Hemorrhage (%)	0 (0.0)	1 (0.9)	N/A
Infection (%)	0 (0.0)	1 (0.9)	N/A
Poor wound healing (%)	0 (0.0)	2 (1.7)	N/A
Lymphocele (%)	0 (0.0)	3 (2.6)	N/A
Thrombosis/occlusion (%)	0 (0.0)	0 (0.0)	N/A
Vascular injury (%)	0 (0.0)	2 (1.7)	N/A
Pseudoaneurysm (%)	0 (0.0)	0 (0.0)	N/A
Lower limb ischemia (%)	0 (0.0)	0 (0.0)	N/A
Femoral artery dissection (%)	0 (0.0)	0 (0.0)	N/A

Postoperative wound disinfection care (times)	1.08 ± 0.39	3.31 ± 0.91	<0.001

Length of hospital stay (days)	5.19 ± 2.26	8.33 ± 3.76	<0.001

Continuous variables were present as median (Q25, Q75) or mean ± SD. *χ*^2^ test for categorical variables and *t*-test or Wilcoxon rank sum test for continuous variables.

**Table 4:** Univariable and multivariable logistic regression analysis of risk factors for postoperative approach procedure-related complications.

Valuables	Univariable		Multivariable	
	*p* value	OR (95% CI)	*p* value	OR (95% CI)
Preoperative factors				
Age (years)	0.031	1.05 (1.00, 1.10)	0.094	1.04 (0.99, 1.12)
Male gender	0.374	1.72 (0.52, 5.70)	—	—
Obesity	<0.001	16.81 (5.03, 56.17)	<0.001	22.26 (4.74, 104.49)
Hypertension	0.167	0.43 (0.13, 1.43)	0.548	0.61 (0.12, 3.07)
Diabetes mellitus	0.440	2.33 (0.27, 19.75)	—	—
Coronary heart disease	0.087	4.10 (0.82, 20.54)	0.420	2.13 (0.34, 13.40)
Hypercholesterolemia	0.060	3.23 (0.95, 10.91)	0.469	1.84 (0.36, 9.52)
Hypoproteinemia	0.130	3.42 (0.70, 17.00)	0.262	3.10 (0.43, 22.31)
Moderate anemia	0.999	—	—	—
Platelet count	0.871	1.01 (0.89, 1.20)	—	—
White blood cell count	0.266	1.00 (0.99, 1.01)	—	—

Intraoperative factors				
Operative time (min)	0.042	1.03 (1.00, 1.07)	0.615	0.98 (0.91, 1.06)
Operative approach procedure time (min)	0.040	1.004 (1.00, 1.07)	0.953	1.00 (0.89, 1.13)
Intraoperative blood loss	0.330	1.03 (0.97, 1.07)	—	—

Stent information				
Lifetech scientific	0.757	0.81 (0.22, 3.02)	—	—
Medtronic	0.581	0.56 (0.07, 4.41)	—	—
MicroPort	0.913	1.12 (0.14, 9.08)	—	—

Sheath diameter				
16Fr	0.102	2.49 (0.83, 7.44)	0.216	2.36 (0.61, 9.20)
18Fr	0.305	0.57 (0.19, 1.68)	—	—
20Fr	0.606	0.67 (0.15, 3.08)	—	—

Group (PPST or FAIT)	0.007	6.05 (1.65, 22.19)	0.080	9.07 (0.77, 107.51)

Those factors *p* < 0.200 in the univariable model were involved in the multivariable model.

## Data Availability

The data of this study will not be shared publicly because they will be applied for further research of this series. But corresponding authors do agree that the data can be shared individually if requested.

## Abbreviations

PPST: Percutaneous presuture technique
FAIT: Femoral artery incision technique
EVAR: Endovascular aortic repair
uTBAD: Uncomplicated type B aortic dissection
TBAD: Stanford type B aortic dissection.
